# A Conditional Protein Degradation System To Study Essential Gene Function in Cryptosporidium parvum

**DOI:** 10.1128/mBio.01231-20

**Published:** 2020-08-25

**Authors:** Hadi H. Choudhary, Maria G. Nava, Brina E. Gartlan, Savannah Rose, Sumiti Vinayak

**Affiliations:** a Department of Pathobiology, College of Veterinary Medicine, University of Illinois at Urbana-Champaign, Urbana, Illinois, USA; b School of Molecular and Cellular Biology, College of Liberal Arts and Sciences, University of Illinois at Urbana-Champaign, Urbana, Illinois, USA; Albert Einstein College of Medicine

**Keywords:** CRISPR/Cas9, conditional system, *Cryptosporidium*, apicomplexan parasite, drug targets, molecular genetics

## Abstract

*Cryptosporidium* spp., protozoan parasites, are a leading cause of global diarrhea-associated morbidity and mortality in young children and immunocompromised individuals. The limited efficacy of the only available drug and lack of vaccines make it challenging to treat and prevent cryptosporidiosis. Therefore, the identification of essential genes and understanding their biological functions are critical for the development of new therapies. Currently, there is no genetic tool available to investigate the function of essential genes in *Cryptosporidium* spp. Here, we describe the development of the first conditional system in Cryptosporidium parvum. Our system utilizes the Escherichia coli dihydrofolate reductase degradation domain (DDD) and the stabilizing compound trimethoprim (TMP) for conditional regulation of protein levels in the parasite. We tested our system on the calcium-dependent protein kinase-1 (CDPK1), a leading drug target in C. parvum. By direct knockout strategy, we establish that *cdpk1* is refractory to gene deletion, indicating its essentiality for parasite survival. Using CRISPR/Cas9, we generated transgenic parasites expressing CDPK1 with an epitope tag, and localization studies indicate its expression during asexual parasite proliferation. We then genetically engineered C. parvum to express CDPK1 tagged with DDD. We demonstrate that TMP can regulate CDPK1 levels in this stable transgenic parasite line, thus revealing the critical role of this kinase in parasite proliferation. Further, these transgenic parasites show TMP-mediated regulation of CDPK1 levels *in vitro* and an increased sensitivity to kinase inhibitor upon conditional knockdown. Overall, this study reports the development of a powerful conditional system that can be used to study essential genes in *Cryptosporidium*.

## Abstract (precis)

Cryptosporidium parvum and Cryptosporidium hominis are leading pathogens responsible for diarrheal disease (cryptosporidiosis) and deaths in infants and children below 5 years of age. There are no effective treatment options and no vaccine for cryptosporidiosis. Therefore, there is an urgent need to identify essential gene targets and uncover their biological function to accelerate the development of new and effective anticryptosporidial drugs. Current genetic tool allows targeted disruption of gene function but leads to parasite lethality if the gene is essential for survival. In this study, we have developed a genetic tool for conditional degradation of proteins in *Cryptosporidium* spp., thus allowing us to study the function of essential genes. Our conditional system expands the molecular toolbox for *Cryptosporidium*, and it will help us to understand the biology of this important human diarrheal pathogen for the development of new drugs and vaccines.

## INTRODUCTION

Diarrhea is a leading global cause of mortality in children under 5 years of age, with 1.7 billion cases and 525,000 deaths annually (https://www.who.int/en/news-room/fact-sheets/detail/diarrhoeal-disease). Large-scale global epidemiological studies have reported the protozoan parasite *Cryptosporidium* (C. parvum and C. hominis) to be the second leading pathogen after rotavirus of diarrhea among children aged 0 to 59 months residing in low-income communities in sub-Saharan Africa and Asia (1, 2). Repeated episodes of *Cryptosporidium* infection and malnutrition in young children have been found to be associated with growth stunting and developmental defects leading to loss of 7.85 million disability-adjusted life years (DALYs) (2, 3). In addition to infecting young children with low immunity, *Cryptosporidium* spp. are important opportunistic pathogens and can be life-threatening in immunocompromised individuals such as those living with HIV/AIDS or organ transplant recipients (4–7). Infection with *Cryptosporidium* occurs through the contamination of water and food sources with oocysts, a parasite stage that is resistant to standard disinfection procedures such as chlorination. Transmission by Cryptosporidium hominis is restricted only to humans (anthroponotic), while Cryptosporidium parvum has both an anthroponotic and zoonotic transmission cycle. C. parvum naturally infects ruminants, especially calves, causing neonatal diarrhea (calf scours), and transmission from animals to humans occurs via contact with infected animals (8). Cryptosporidiosis is also a major public health concern in developed countries, and frequent outbreaks occur each year due to oocyst contamination of recreational water facilities such as swimming pools and water parks (9, 10).

There are no options for effective treatment and no vaccines for the prevention of cryptosporidiosis. The only U.S. FDA-approved drug nitazoxanide is not effective in immunocompromised individuals and has poor efficacy in malnourished children (11, 12). Due to the urgent need for development of new and effective drugs, several studies have focused on identifying anticryptosporidial compounds or small molecule inhibitors. Using phenotyping screens and drug repurposing approaches, potent compounds have been identified that are effective in killing C. parvum *in vitro* and in animal models of infection (13–18). However, the target of these compounds and the biological processes they disrupt during the parasite’s life cycle are not known. On the other hand, targeted drug screening efforts in C. parvum that are based on conserved targets in related apicomplexan parasites such as Toxoplasma gondii and Plasmodium falciparum have generated lead compounds that show promise for clinical development (19–21). Calcium-dependent protein kinase-1 (CDPK1) is one of the leading drug targets in C. parvum. Potent bumped kinase inhibitors (BKIs) targeted against CDPK1 have been found to be efficacious in immunocompromised interferon gamma knockout (IFN-γ KO), immunodeficient SCID-gamma, and neonatal mouse models, as well as in the natural calf model for C. parvum infection (21–24). In T. gondii, *cdpk1* is an essential gene that is required for calcium-dependent release of proteins stored in apical secretory organelles (micronemes) that in turn controls parasite motility, host cell invasion, and exit from the cell (25). In P. falciparum and Plasmodium berghei, *cdpk4* (ortholog of the *cdpk1* gene) plays essential functions in male gametogenesis and mosquito transmission (26–28). However, the essentiality of *cdpk1* for C. parvum and the biological functions it regulates in the parasite are not known.

The advent of molecular genetics for *Cryptosporidium*, new mouse models of infection for drug efficacy studies and vaccine development, and improved methods for long-term cultivation of the parasite have greatly enhanced the pace of research (29–32). Despite this progress, a critical barrier in *Cryptosporidium* research is the lack of a tool to study the function of essential genes vital for parasite survival. Development of conditional strategies that control expression at the gene (inducible Cre, diCre, Flp recombinases), transcriptional (tetracycline-on/off inducible systems), post-transcriptional (ribozymes), and post-translational levels (protein destabilizing domain FKBP-12 regulated by shield-1, Escherichia coli dihydrofolate reductase degradation domain regulated by trimethoprim, auxin-inducible domain) in other parasites have profoundly transformed the understanding of their biology (33–41). Regulation at the post-translational level allows a more rapid regulation, since the control acts directly on the synthesized protein compared to other approaches. Due to its rapid kinetics and tight regulation, the E. coli dihydrofolate reductase (ecDHFR)-based degradation domain system has been successfully adapted to study functions of essential proteins in apicomplexan parasites (40–42). This system involves tagging a gene of interest with the DDD, resulting in a fusion protein that is unstable, leading to its degradation by the proteasome. Addition of the folate analog trimethoprim stabilizes the fusion protein, thus protecting it from degradation.

Here, we report the development of a robust conditional system to study essential gene function in C. parvum. This new genetic system adapts the E. coli dihydrofolate reductase degradation domain (DDD) and the small compound trimethoprim (TMP) for post-translational regulation of protein levels in the parasite (42). We utilized the CRISPR/Cas9 system and immunocompromised mouse model of infection to genetically engineer C. parvum to express this degradation domain and regulate levels of CDPK1 *in vivo* using TMP, thus revealing the critical role of this kinase in parasite proliferation. Moreover, the knockdown of CDPK1 *in vitro* led to increased sensitivity of C. parvum to the bumped kinase inhibitor that selectively targets CDPK1. Overall, we describe the development of the first conditional system for C. parvum that allows investigation of essential gene function during the life cycle of this parasite. This powerful system can be applied to study other essential genes and validate therapeutic targets for cryptosporidiosis.

## RESULTS

### CDPK1 is essential for C. parvum survival.

**FIG 1:**
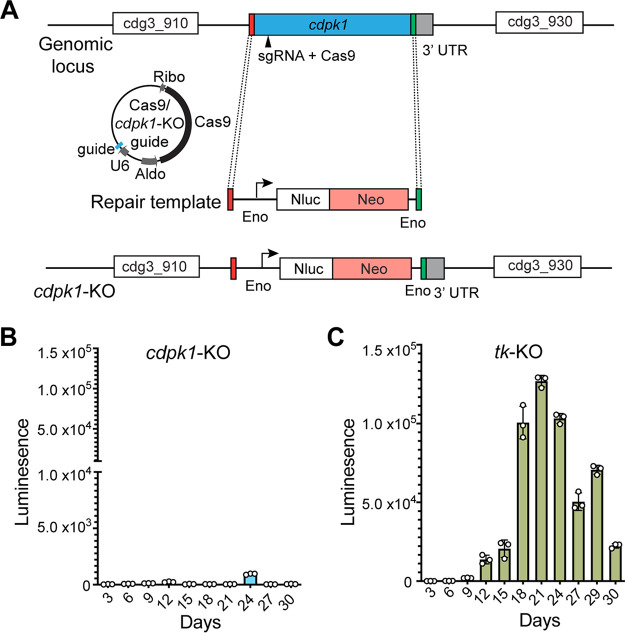
CDPK1 is essential for parasite survival. (A) Schematic showing *cdpk1* locus, location of the single guide RNA (sgRNA) and Cas9-induced DNA break, Cas9/*cdpk1*-KO guide plasmid, and the repair template for homologous generation. Nluc, nanoluciferase; Neo, neomycin resistance marker; Eno, enolase promoter and 3′ UTR; Aldo, aldolase promoter; Ribo, ribosomal 3′UTR. (B and C) Fecal luminescence measurements from mice infected with sporozoites transfected with Cas9/*cdpk1*-KO guide and repair DNA (B) and Cas9/*tk*-KO guide and repair DNA (C). Data points are means ± standard deviations (SD) (error bars) for three technical replicates. Representative data from two independent experiments are shown.

To investigate the essentiality of CDPK1 in parasite survival, we attempted to delete the *cdpk1* gene. Since *Cryptosporidium* spp. are haploid (the only exception being the short diploid zygote stage upon fertilization) and both asexual and sexual stages occur in a single host, disruption of an essential gene at any stage would be lethal for the parasite. We used the CRISPR/Cas9 system to introduce a double-stranded DNA break in the *cdpk1* gene using a specific knockout guide RNA sequence (*cdpk1* knockout [*cdpk1*-KO] guide). We attempted to repair the break via double homologous recombination using a linear repair DNA containing 50-bp homologous sequence 5′ and 3′ of the cut site, and an Eno-Nluc-Neo-Eno (nanoluciferase reporter-neomycin resistance marker flanked by the C. parvum enolase promoter and 3′ enolase untranslated region [UTR]) cassette (Fig. 1A).

We delivered sporozoites transfected with the Cas9/*cdpk1*-KO guide and repair DNA to 5-week-old IFN-γ KO mice (*n* = 4) and provided paromomycin to mice in drinking water to select for neomycin-resistant parasites. As a control, we also attempted to knock out the thymidine kinase (*tk*) gene that we previously reported to be non-essential for parasite survival (29). We measured luminescence on pooled fecal material collected from *cdpk1*-KO mouse cages every 3 days to monitor growth and emergence of resistant parasites and found no increase in luminescence over time (Fig. 1B). In parallel, fecal luminescence measurements from *tk*-KO mouse cages showed the emergence and rise of transgenic parasites (Fig. 1C). Multiple attempts to knock out the *cdpk1* gene were unsuccessful, while the *tk* gene could be deleted in every experiment, suggesting that *cdpk1* is essential for parasite survival.

### Endogenous epitope tagging of C. parvum CDPK1 reveals its role in asexual proliferation.

**FIG 2:**
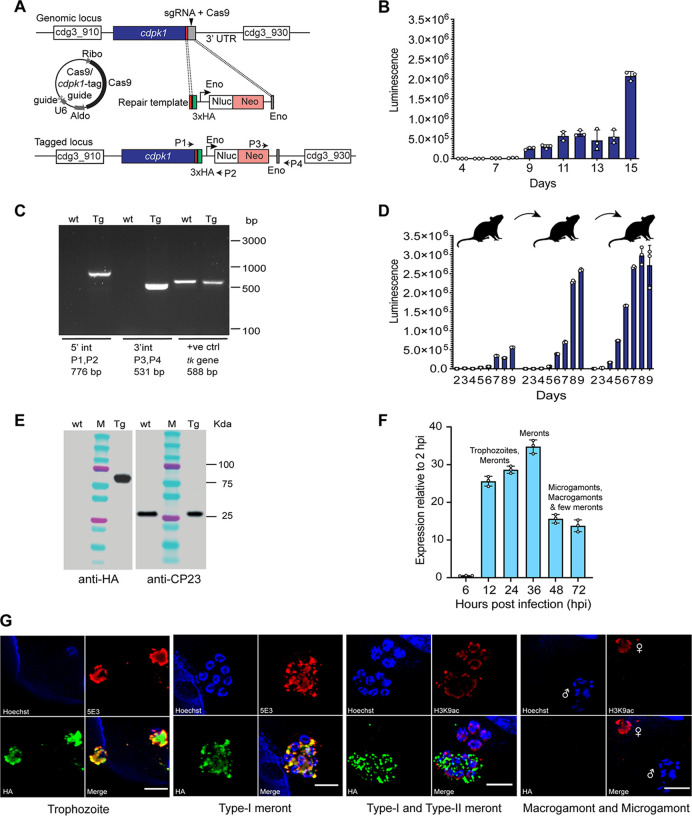
Endogenous epitope tagging of *cdpk1*, expression and localization. (A) Schematic showing *cdpk1* locus, location of the single guide RNA (sgRNA) and Cas9-induced DNA break in the 3′ UTR, Cas9/guide plasmid, and the repair template for homologous recombination. Triple hemagglutinin epitope tag (3xHA) and 50-bp flanking regions for homologous recombination are shown. P1, P2, P3, and P4 are primers used to verify genomic integration events. Sequences of these primers are provided in Table S1 in the supplemental material. (B) Fecal luminescence measurements from mice infected with sporozoites transfected with Cas9/*cdpk1*-tagging guide and repair DNA. Means ± SD (error bars) for three technical replicates are shown. (C) PCR of the transgenic CDPK1-HA tagged parasites confirming the correct 5′ (P1/P2) and 3′ (P3/P4) integration (int) events. Fecal genomic DNAs from wild-type (wt) and transgenic (Tg) parasites were used as the template. The thymidine kinase (*tk*) gene was amplified as a positive control (+ve ctrl) using *tk*-specific primers. (D) Fecal luminescence measurements (three passages) from IFN-γ KO mice infected with CDPK1-HA transgenic oocysts. Means ± SD for three technical replicates are shown. (E) Western blot showing CDPK1 expression using anti-HA antibody on wild-type (wt) and transgenic (Tg) parasites. CP23 was used as a loading control. (F) Expression of *cdpk1* at indicated time points of infection of HCT-8 cells with wild-type oocysts. Data points are means ± SD for three technical replicates. Trophozoites, meronts, merozoites, and sexual stages (microgamonts and macrogamonts) seen at different time points of HCT-8 infection are indicated. (G) Immunofluorescence assay and super-resolution microscopy showing CDPK1 expression in trophozoites and type-I meronts using anti-HA (green) and 5E3 (red) antibodies after 24 h of HCT-8 infection. CDPK1 is expressed only in the mature type-I meront (8-nuclei stage) and not in type-II meront (4-nuclei stage) as shown using antibodies against HA (green) and H3K9ac (red) after 48 h of HCT-8 infection. No expression is seen in the male (microgamont) and female (macrogamont) sexual stages at 48 hpi. Parasite nuclei were counterstained with Hoechst (blue). Representative data from two independent experiments are shown. Bars, 2 μm.

To determine whether the *cdpk1* gene locus is amenable to genetic recombination and to understand the cellular localization of the expressed CDPK1 protein, we generated a stable transgenic parasite line with a triple hemagglutinin (3xHA) epitope tag at the C-terminus of CDPK1 (Fig. 2A). The guide sequence used was located in the 3′ untranslated region (3′UTR) of the gene, 28 bp after the stop codon. The repair contained 50-bp regions of homology upstream of the stop codon, the 3xHA-Eno-Nluc-Neo-Eno, and 50-bp regions of homology downstream of the protospacer adjacent motif (PAM) to avoid cutting of the repair DNA. Measurement of fecal Nluc from mice infected with transfected sporozoites revealed an increase in luminescence on day 9 post-infection with a peak in reading on day 15, thus indicating the generation of transgenic CDPK1-HA-tagged parasites (Fig. 2B). PCR amplification of fecal genomic DNA confirmed the correct 5′ and 3′ integration events (Fig. 2C). No PCR amplification was observed in the wild-type DNA for the 5′ and 3′ integration PCR. Purified oocysts from the fecal material were passaged three times into naive IFN-γ KO mice to generate a stable transgenic line and to obtain enough fecal material for further experiments. As shown in Fig. 2D, passaging of oocysts purified from the first passage resulted in an ∼4.6-fold increase in fecal luminescence at day 9 post-infection. The third passage showed almost a similar infection dynamic pattern as the second passage. Western blotting of lysed transgenic parasites using anti-HA antibody confirmed the expression of the CDPK1, and the expected 65-kDa tagged protein was detected (Fig. 2E). Wild-type (wt) parasites without the HA tag were used as a negative control. No band was detected using the anti-HA antibody for the wt parasites (Fig. 2E). The CP23 antibody that detects an ∼27-kDa C. parvum glycoprotein was used as a loading control (43).

10.1128/mBio.01231-20.3 TABLE S1 Sequences of oligonucleotides used in this study. Download Table S1, DOCX file, 0.02 MB. Copyright © 2020 Choudhary et al. 2020 Choudhary et al. This content is distributed under the terms of the Creative Commons Attribution 4.0 International license.

To examine the expression dynamics of *cdpk1*, we infected HCT-8 host cells with wild-type oocysts for different lengths of time, followed by reverse transcription-quantitative PCR (RT-qPCR) (Fig. 2F). A high level of expression of *cdpk1* was observed at 12 to 36 h post-infection (hpi), indicating that this gene is expressed during asexual merogony (Fig. 2F). Asexual stages (trophozoites, meronts, merozoites) have been reported to occur in cell culture at 12 to 36 hpi, followed by predominantly sexual stages and few meronts at 48 hpi (44, 45). To visualize the localization of CDPK1, we performed immunofluorescence assays and super-resolution microscopy of CDPK1-HA transgenic parasites. CDPK1 was found to be expressed in the trophozoite and meront stages, thus confirming the expression of this kinase during asexual proliferation (Fig. 2G). CDPK1 was located in the merozoites, as detected by the 5E3 antibody that has been previously shown to recognize individual merozoites and their apical poles (46) (Fig. 2G). The expression of CDPK1 in the meronts was seen even during the late stages (48 hpi) of the parasite cycle. Interestingly, CDPK1 was expressed only in the mature type-I meront that harbors eight merozoites, but not in the type-II meront (four-merozoite stage) (Fig. 2G). Although by qPCR we observed gene expression at later time points when sexual stages are reported to develop, there was no expression of CDPK1 in mature sexual stages at 48 hpi. The macrogamont (female) was visualized by the dense staining of its nucleus using antibody against histone H3 acetylated at lysine 9 (H3K9Ac), and the microgamont (male) was recognized by 16 bullet-shaped nuclei of the microgametes (Fig. 2G). However, there are no established markers to track early or developing microgamonts and distinguish them from type-I meronts. Based on the high expression and localization of CDPK1 seen in type-I meronts, our results suggest that CDPK1 is required during the asexual replicating stages of the parasite life cycle.

### Conditional protein degradation system for C. parvum.

**FIG 3:**
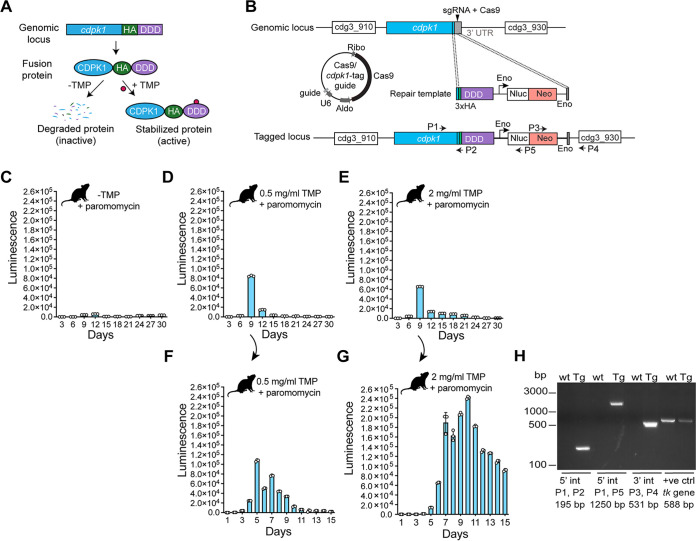
Generation of conditional protein degradation system. (A) Schematic of the conditional system for regulating CDPK1 protein. (B) Strategy for tagging the *cdpk1* gene with 3xHA and DDD. Location of the single guide RNA (sgRNA) and Cas9-induced DNA break in the 3′ UTR, Cas9/guide plasmid, and the repair template for homologous recombination are shown. P1, P2, P3, P4, and P5 are primers used to verify genomic integration events. Sequences of these primers are provided in Table S1. (C to E) Fecal luminescence measurements from mice infected with transfected sporozoites, kept in cages with no TMP (C) or with 0.5 mg/ml TMP (D) or 2 mg/ml TMP (E) in drinking water. (F and G) Passaging of fecal material into naive IFN-γ KO mice and luminescence measurements from those mice, kept in cages with 0.5 mg/ml TMP (F) and 2 mg/ml TMP (G) in drinking water. (H) PCR of the transgenic CDPK1-HA-DDD-tagged parasites confirming the correct 5′ (P1/P2 and P1/P5) and 3′ (P3/P4) integration events. The thymidine kinase (*tk*) gene was amplified as a positive control using *tk*-specific primers. Fecal genomic DNA from wild-type (wt) and transgenic (Tg) parasites were used as the template. In panels C to G, data points are means ± SD for three technical replicates. Representative data from two independent experiments are shown. All infected mice were given paromomycin (16 mg/ml) during the entire course of the experiment.

To develop a conditional system for C. parvum based on the E. coli DHFR degradation domain (DDD), we took advantage of the natural resistance of C. parvum to antifolates, CRISPR/Cas9 editing, immunocompromised mouse model of infection, and the inexpensive compound TMP with favorable pharmacological properties (29, 47). We devised a strategy that employed the CRISPR/Cas9 system to genetically edit the essential *cdpk1* gene locus by appending 3xHA and DDD at its 3′ end (Fig. 3A and B). We expected that the fusion protein (CDPK1-HA-DDD) would be unstable, leading to its degradation by the proteasome. However, addition of the antifolate TMP would allow stabilization of the fusion protein given the high affinity of TMP to DDD, thus preventing degradation of the protein (Fig. 3A).

To generate a stable transgenic C. parvum line expressing the CDPK1-HA-DDD, we used the same *cdpk1* tagging guide but a different repair DNA. We cloned the DDD fragment downstream of the 3xHA in the CplicHA_3_-Eno-Nluc-Neo-Eno vector to generate the 3xHA-DDD-Eno-Nluc-Neo-Eno construct. This construct was used as a template to generate a linear repair DNA containing 50-bp regions of homology arms upstream of the stop codon and downstream of the PAM motif (Fig. 3B). The sporozoites transfected with the *cdpk1* tagging guide and the repair DNA were delivered to IFN-γ KO mice by oral gavage. Mice were administered water containing paromomycin for selection of neomycin-resistant parasites and TMP for conditional regulation of CDPK1. Mice infected with transfected parasites that were administered only paromomycin and no TMP did not show any increase in fecal luminescence over the 30-day period (Fig. 3C). On the other hand, mice that were infected with transfected sporozoites and administered a combination of paromomycin (16 mg/ml) and TMP (0.5 mg/ml or 2 mg/ml) showed an increase in luminescence on day 9, indicating the emergence of selected parasites, but then the infection gradually tapered off over time (Fig. 3D and E). The fecal material from day 9 from both the 0.5- and 2-mg/ml TMP mouse cages were then used to infect naive IFN-γ KO mice and treated again with TMP and paromomycin (Fig. 3F and G). An early infection was seen on day 6, but increased parasite growth over the next 15 days was observed in mice that received 2 mg/ml TMP compared to mice treated with 0.5 mg/ml TMP (Fig. 3F and G). The robust infection observed in mice treated with 2 mg/ml TMP strongly suggests the generation of viable transgenic parasites in which the CDPK1 levels were stabilized by TMP (Fig. 3G). We validated the stable transgenic CDPK1-HA-DDD line by performing PCR on genomic DNA from fecal material collected from infected mice treated with 2 mg/ml TMP. The 195-bp and 1,250-bp bands confirmed the correct 5′ integration event, while the 531-bp band indicated the expected 3′ integration event (Fig. 3H). We also investigated whether daily oral gavage with 2 mg/ml TMP instead of administering TMP in drinking water would allow generation of the transgenic CDPK1-HA-DDD line. Oral gavage with TMP (but paromomycin in drinking water) led to an increase in fecal luminescence on day 10 post-infection, indicating the emergence of paromomycin-resistant parasites (see Fig. S1 in the supplemental material). This indicates that both routes of TMP administration are efficient in generating a conditional knockout line. Since administering TMP in drinking water is less tedious than daily oral gavage, we decided to use this method for all further experiments.

10.1128/mBio.01231-20.1 FIG S1 Fecal luminescence measurements from mice infected with transfected sporozoites to create stable CDPK1-HA-DDD transgenic parasites. Mice were administered paromomycin (16 mg/ml) in drinking water without TMP (A) or given TMP (2 mg/ml) by daily oral gavage (B). Download FIG S1, TIF file, 0.7 MB. Copyright © 2020 Choudhary et al. 2020 Choudhary et al. This content is distributed under the terms of the Creative Commons Attribution 4.0 International license.

### TMP regulates CDPK1 levels and parasite growth *in vivo*.

**FIG 4:**
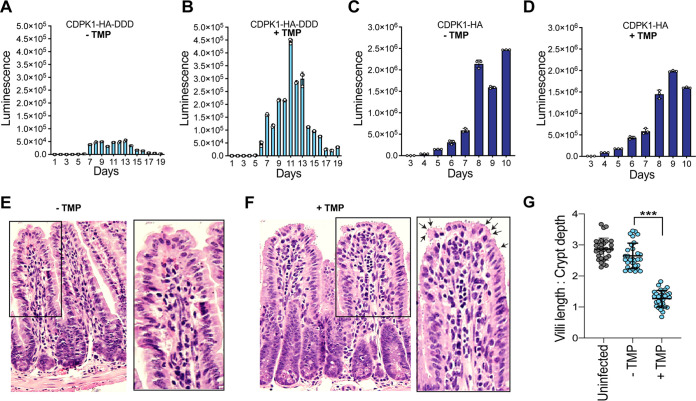
Conditional regulation of CDPK1 protein *in vivo*. (A and B) Fecal luminescence measurements from IFN-γ KO mice infected with CDPK1-HA-DDD stable transgenic oocysts without TMP (A) and with TMP (B). (C and D) Fecal luminescence measurements from IFN-γ KO mice infected with CDPK1-HA stable transgenic oocysts without TMP (C) and with TMP (D). (E and F) Histology of distal part of small intestine from mice infected with CDPK1-HA-DDD stable transgenic oocysts without TMP (E) and with TMP (F). Black arrows indicate C. parvum infection. An enlarged section of the villi is shown for each panel. (G) Measurement of villus length and crypt depth ratio for 30 villi from uninfected and CDPK1-HA-DDD infected mice. All infected mice were given paromomycin (16 mg/ml) during the entire course of the experiment. In panels A to D, data points are means ± standard deviation (SD) for three technical replicates. ***, *P* < 0.001.

We sought to investigate whether CDPK1-HA-DDD can be regulated *in vivo* using TMP. Mice infected with these parasites and administered TMP in drinking water showed a significant increase in fecal luminescence (as a proxy for parasite growth) starting day 4 post-infection, with a significant 9.5-fold increase in parasite burden on day 11, compared to mice that were not treated with TMP (unpaired *t* test, *P* < 0.000001) (Fig. 4A and B). This suggests that stabilization of CDPK1 levels by TMP results in increased parasite proliferation, while absence of TMP results in parasite lethality. We further confirmed that the increased parasite growth is due to regulation of C. parvum CDPK1 levels and that continuous administration of TMP is not toxic for parasite growth by infecting mice with the CDPK1-HA strain and monitoring growth in the absence or presence of TMP. Although the CDPK1-HA transgenic line showed higher infection rate than the CDPK1-HA-DDD, there was no significant change in luminescence for the parasites propagated in the absence or presence of TMP (Mann-Whitney test, two-tailed *P* = 0.96) (Fig. 4C and D).

We also determined whether CDPK1 stabilization translates into the increased C. parvum proliferation in the small intestine of infected mice. We hypothesized that CDPK1 degradation would result in parasite lethality and decreased parasite burden in the intestine. We infected two new cages of IFN-γ KO mice with CDPK1-HA-DDD and treated one group of mice with 2 mg/ml TMP in drinking water, while no TMP was administered to the other group. As expected, we found a significant increase in parasite growth by fecal luminescence in mice in the TMP-treated cage (Fig. S2). We sacrificed mice (*n* = 3) from both cages on day 11 post-infection and performed histological examination of the distal part of the small intestine. Mice infected with CPDK1-HA-DDD with no TMP administration revealed the absence of C. parvum on the villus surface and an architecturally intact intestinal mucosa, demonstrating that degradation of the essential CDPK1 results in parasite lethality (Fig. 4E). In contrast, a high C. parvum infection with characteristic villus atrophy (blunting of villi), and crypt hyperplasia in TMP-treated mice was observed, indicating that stabilization of CDPK1 results in increased parasite proliferation and intestinal abnormalities (Fig. 4F). Quantitative measurements of the villus length and crypt depth revealed a significant decrease in ratio (*P* < 0.0001, one-way analysis of variance [ANOVA] with multiple comparisons) in the TMP-treated versus non-TMP-treated and uninfected mice, indicating increased flattening of villi and enlargement of crypt due to high C. parvum burden (Fig. 4G). There was no aberrant change in the intestinal mucosa of mice infected with CPDK1-HA-DDD transgenic parasites with no TMP administration compared to uninfected control mice (Fig. 4G).

10.1128/mBio.01231-20.2 FIG S2 Fecal luminescence measurements from mice (*n* = 5) infected with CDPK1-HA-DDD stable transgenic oocysts. Mice were administered paromomycin (16 mg/ml) without TMP (A) and with TMP (B) in drinking water. Mice (*n* = 3) were sacrificed on day 11 post-infection for histology. Download FIG S2, TIF file, 0.5 MB. Copyright © 2020 Choudhary et al. 2020 Choudhary et al. This content is distributed under the terms of the Creative Commons Attribution 4.0 International license.

### TMP regulates CDPK1 levels *in vitro*.

**FIG 5:**
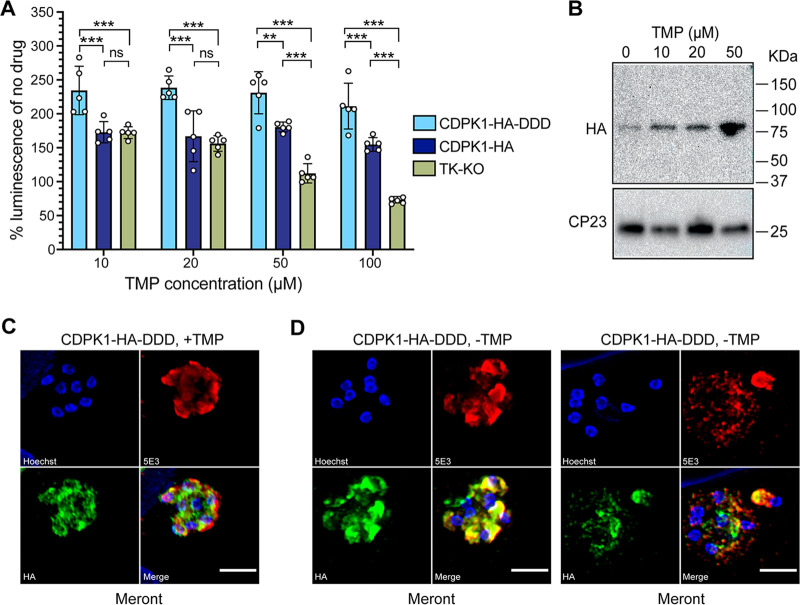
Conditional regulation of CDPK1 protein *in vitro*. (A) Measurement of luciferase activity of CDPK1-HA-DDD (sky blue), CDPK1-HA (dark blue), and TK-KO (green) parasites incubated with different concentrations of TMP after 48 h of infection on HCT-8 cells. Data points are means ± SD for five technical replicates. Representative data from two independent experiments are shown. ***, *P* < 0.001; **, *P* = 0.002; ns, not significant. (B) Western blot showing CDPK1 levels in CDPK1-HA-DDD transgenic parasites grown in the absence or presence of TMP. Anti-HA antibody was used to probe for CDPK1, and anti-CP23 antibody was used as a loading control. (C and D) Immunofluorescence assay and super-resolution microscopy of CDPK1-HA-DDD parasites grown for 24 h on HCT-8 cells in the presence of 20 μM TMP (+TMP) (C) and absence of TMP (-TMP) (D). Anti-HA (green) and 5E3 (red) antibodies were used for staining. Parasite nuclei were counterstained with Hoechst (blue). Bars, 2 μm.

We further tested the ability of CDPK1-HA-DDD transgenic parasites to regulate protein levels *in vitro*. We infected HCT-8 cells with 5,000 oocysts from stable transgenic lines (CDPK1-HA-DDD, CDPK1-HA, and TK-KO), and incubated them with or without TMP for 48 h. After 48 h of infection, measurement of luminescence revealed increased growth of CDPK1-HA-DDD in the presence of TMP (Fig. 5A). Although the CDPK1-HA parasites could also grow in the presence of TMP due to their inherent resistance to antifolate, they displayed significantly reduced level of growth compared to CDPK1-HA-DDD at all TMP concentrations (Fig. 5A). As expected, the TK-KO parasites showed significant reduction in growth at 50 and 100 μM TMP concentrations compared to CDPK1-HA-DDD and CDPK1-HA parasites, confirming that the loss of TK results in the sensitivity of C. parvum to antifolate. We determined CDPK1 protein levels in CDPK1-HA-DDD parasites grown in the presence and absence of TMP by Western blotting (Fig. 5B). There was a 4-fold increase in CDPK1 expression levels (after normalization to loading control) at 10 μM TMP compared to the parasites grown in the absence of TMP. As expected, 50 μM TMP concentration led to increased stabilization of CDPK1, resulting in a 15-fold increase in protein level compared to the no-TMP sample (Fig. 5B). For a functional assessment on expression and localization of CDPK1 in the asexual stages upon conditional knockdown, we performed super-resolution microscopy of CDPK1-HA-DDD parasites grown in the presence or absence of TMP. CDPK1-HA-DDD parasites grown in the presence of 20 μM TMP showed localization of CDPK1 in the type-I meront (Fig. 5C). In the absence of TMP, only a few parasites showed expression of CDPK1 due to knockdown of CDPK1. These CDPK1 knockdown parasites showed condensed staining at the apical end of the merozoites or very weak diffused staining in the meront, thus indicating the role of this kinase in merozoite development and asexual proliferation (Fig. 5D).

### Transgenic parasites with reduced CDPK1 levels are highly sensitive to BKI-1294.

**FIG 6:**
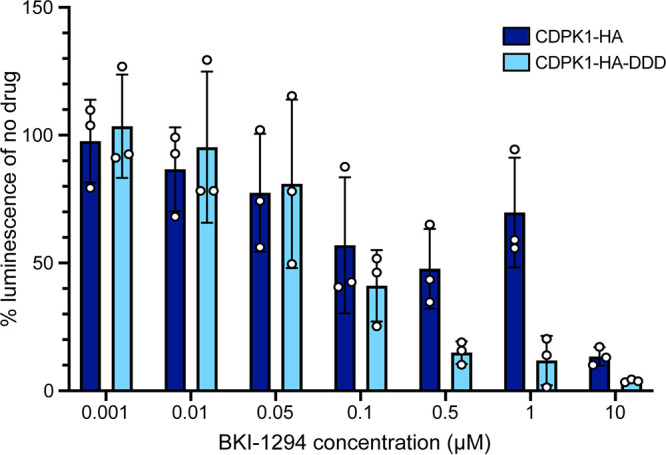
Bar plot showing increased sensitivity of CDPK1-HA-DDD parasites to BKI-1294. Measurement of luciferase activity of CDPK1-HA-DDD (sky blue) and CDPK1-HA (dark blue) parasites grown in the presence of different concentrations of BKI-1294. Data points are means ± SD for three technical replicates. Representative data from two independent experiments are shown.

Previous studies have demonstrated that the bumped kinase inhibitor BKI-1294 targeted against CDPK1 is highly effective in inhibiting C. parvum growth *in vitro* (24, 48). We hypothesized that conditional knockdown of CDPK1 protein levels *in vitro* would result in increased susceptibility of the parasite to the action of BKI-1294. We infected HCT-8 cells with oocysts from CDPK1-HA-DDD (from mice treated with TMP) and CDPK1-HA transgenic lines and incubated them without TMP but with increasing concentrations of the BKI-1294. Luminescence was quantified after 48 h of infection, and the 50% effective concentration (EC_50_) of BKI-1294 was determined. As expected, the CDPK1-HA-DDD showed increased sensitivity to BKI-1294 (EC_50_ = 104 nM) compared to CDPK1-HA transgenic (EC_50_ = 724 nM) (Fig. 6). The 7-fold shift in EC_50_ strongly suggests that knockdown of CDPK1 results in increased sensitivity of C. parvum to the action of the BKI-1294, resulting in further inhibition of parasite growth.

## DISCUSSION

The development of a conditional system is critical to interrogate the function of essential genes in *Cryptosporidium* spp. Functional characterization of essential genes is important for the development and validation of drug targets. In this study, we report the development of the first conditional system, based on the dihydrofolate reductase (DHFR) degradation domain, in C. parvum. We applied this tool to conditionally regulate the levels of the essential CDPK1 protein *in vivo* and *in vitro*. Using this system, we show that *cdpk1* is indispensable for the parasite survival and that stabilization of CDPK1 protein is vital for parasite growth.

Major factors that facilitated the development of this system for C. parvum were its non-toxicity to antifolates, low cost of the TMP compound compared to shield-1 (used in DDFKBP-12 system), and the good pharmacological characteristics of TMP for safe and prolonged use in animals (42). In P. falciparum, toxicity of TMP was overcome by engineering parasites containing a human DHFR (hDHFR) marker integrated into a non-essential gene and using the resistant parasites to generate a transgenic line for tagging an essential gene with DDD (40). In contrast, C. parvum is naturally resistant to antifolates, since in addition to DHFR, it has another alternative mechanism for nucleotide synthesis through thymidine kinase (TK), thus rendering DHFR dispensable (29, 49). Thus, the inherent resistance of C. parvum to the antifolates worked to our advantage for application of TMP *in vivo* to create a conditional transgenic parasite. To optimize the DDD system for C. parvum, we administered doses of TMP that have been found to work efficiently in other *in vivo* studies. In rodent models, a range of 0.25 to 2.0 mg/ml TMP in drinking water has been shown to regulate temporal and dose-dependent expression of DDD-tagged fusion proteins in the brain, neuronal tissues, and eye (42, 50, 51). Daily change of water containing TMP or change of water every 3 days or weekly, with prolonged treatment for 3 to 6 weeks for DDD-mediated protein regulation *in vivo* has been reported (42, 50, 52, 53). Although we could generate transgenic CDPK1-HA-DDD parasites using a TMP dose of 0.5 mg/ml, we found a more robust infection upon administering 2 mg/ml TMP in drinking water. This could be due to the wash out or excretion of TMP *in vivo*, and a continuous higher dose of TMP led to increased stabilization of the CDPK1 and increased parasite proliferation.

In the immunocompromised mouse model of infection, C. parvum completes the entire life cycle (both the asexual and sexual stages) in the intestine resulting in shedding of oocysts in feces. A key advantage of applying the DDD approach in the mouse infection model is that it enabled us to quantify the effect of CDPK1 knockdown on parasite growth over multiple life cycles of C. parvum. We found a significant increase in C. parvum growth for CDPK1-HA-DDD parasites grown in the presence of TMP by fecal luminescence assay and high parasite burden on the villus surface, thus revealing the essential role of this kinase in parasite proliferation. Although we did not see any *Cryptosporidum* infection on the villus surface or changes in small intestine architecture in the absence of TMP, we did observe low fecal luminescence readings possibly due to lingering Nluc protein that eventually withered away over subsequent infection cycles. However, *in vitro*, using luciferase assay, we could detect only a 2.3-fold increase in the growth of the CDPK1-HA-DDD transgenic parasite grown in the presence of TMP compared to the no-TMP control. This difference in growth between *in vitro* and *in vivo* is not surprising, since in the HCT-8 cell culture system, parasite growth stalls due to a block in gamete fusion, resulting in no oocyst formation and thus lack of multiple cycles of reinfection and proliferation (44). Nevertheless, we could detect an upregulation (4- to 15-fold increase) of CDPK1 protein levels by Western blotting in parasites grown in increasing concentrations of TMP. The increased stabilization of CDPK1 observed *in vitro* in the presence of increasing concentrations of TMP corroborates with reported studies in other apicomplexan parasites. A 0.5- to 4-fold increase in asparagine repeat protein (Rpn6) levels over the no-TMP sample in dose-response Western blots was reported in P. falciparum (40). An *in vivo* study showed a 2-fold change in inhibitor of cysteine protease (ICP)-DDD fusion protein level in Plasmodium yoelii, upon administration of TMP for 48 h, by Western blotting (41). Lower levels of protein degradation *in vitro* with more pronounced changes in parasite proliferation have also been seen using shield-1 and the FKBP12-DD approach. For example, removal of shield-1 showed a >2-fold change in P. falciparum calcineurin B levels upon propagation of asexual stages of the parasite in red blood cells, but parasite proliferation was inhibited from one cell cycle to the next cycle by ∼70% (54).

There may be several underlying reasons for observing growth inhibition but not a complete loss of CDPK1 protein *in vitro* in the absence of TMP. The high turnover rate of the protein as well as our inability to look at multiple life cycles of parasite growth in the HCT-8 cell culture model could have resulted in the incomplete CDPK1 degradation seen on the Western blot. It is also possible that CDPK1 levels are post-transcriptionally or post-translationally regulated and the DDD system may be perturbating this regulation, thus resulting in incomplete knockdown of CDPK1 in the absence of TMP or aberrant expression in sexual developmental stages during the parasite cycle. In other apicomplexan parasites, post-translational modification such as myristoylation of signaling effectors has been demonstrated to play critical roles in cellular localization and regulating key parasite functions such as microneme secretion, invasion, and egress (39, 55–57). Since there is a predicted N-myristoylation site in C. parvum CDPK1 (58), it is possible that post-translational modification of this protein may be required for proper localization and function. To further understand the mechanistic role of CDPK1 in parasite proliferation, it will be interesting to identify the target protein(s) that it phosphorylates and interacting partners. However, the limited parasite propagation in HCT-8 cells currently makes it challenging to specifically isolate C. parvum meronts in high numbers for pulldown assays and phosphoproteomics experiments. With recent advances in intestinal organoid systems, enteroid culture, and air-liquid interface cultures derived from mouse intestinal epithelial stem cells, it may be possible in the future to scale-up parasite yields to perform these assays (30–32, 59).

In conclusion, we establish a robust conditional system for studying essential gene function in C. parvum. This provides a critical tool that will greatly facilitate investigation of functions of other essential genes in the parasite and validate targets for anticryptosporidial drug development.

## MATERIALS AND METHODS

### Animal ethics statement.

All animal studies and procedures described in this study were approved by the Institutional Animal Care and Use Committee (IACUC) of the University of Illinois Urbana-Champaign under protocol 17188. Age- and sex-matched interferon gamma knockout (IFN-γ KO) mice were used in the experiments. Breeder pairs of IFN-γ KO mice (B6.129S7-*Ifng^tm1Ts^*/J) were purchased from the Jackson Laboratory, and a mouse colony was maintained at our in-house animal breeding facility. Mice (4 to 6 weeks old) were randomly assigned to groups (*n* = 4 or *n* = 5) for generating and passaging stable transgenic C. parvum lines. During the infection experiments, mice were monitored for weight loss, fur ruffling, hunched posture, and inactivity. Mice showing a weight loss of ≥ 15% were euthanized.

### Cloning of guide sequences in Cas9 vector and generation of repair DNA.

Guide RNA sequences targeting the *cdpk1* gene (cgd3_920) or 3′ untranslated region (3′UTR) were cloned into the C. parvum Cas9/guide vector as described previously (29, 60). In this vector, the human codon-optimized Streptococcus pyogenes Cas9 (hSpCas9) gene is driven by the C. parvum aldolase promoter (Aldo) and ribosomal (Ribo) 3′UTR, and the 20-bp guide sequence is under the control of the C. parvum U6 RNA polymerase III promoter. The guide sequences contained on the complementary oligonucleotides were annealed and cloned into the BbsI restriction sites downstream of the C. parvum U6 promoter. A linear repair DNA for replacement of the *cdpk1* gene with the Eno-Nluc-Neo-Eno (nanoluciferase reporter-neomycin resistance marker flanked by the C. parvum enolase promoter and 3′ enolase UTR) cassette was generated by performing a PCR using CplicHA_3_-Eno-Nluc-Neo-Eno vector as a template. The primers OH_cdpk1_F and OH_cdpk1_R used for PCR amplification contained 50-bp region of homology upstream of *cdpk1* start codon and 50-bp region of homology downstream of the stop codon. For tagging the *cdpk1* gene with a triple hemagglutinin (3xHA) epitope tag, OH_CDPK1_HA3_tagF and OH_CDPK1_HA3_tagR primers were used for creating a repair DNA using CplicHA_3_-Eno-Nluc-Neo-Eno as the template. A repair plasmid for integration of the DDD at the 3′ end of the *cdpk1* gene was constructed by amplifying the 477-bp DDD from the pGDB vector (40). The DDD sequence was inserted downstream of the 3xHA epitope tag into the CplicHA_3_-Eno-Nluc-Neo-Eno vector using Gibson assembly. An overhang PCR on the CplicHA_3_-DDD-Eno-Nluc-Neo-Eno plasmid using OH_CDPK1_HA3_tagF and OH_CDPK1_HA3_tagR primers was performed to generate a linear repair DNA. The primers used for guide cloning, overhang PCRs, and Gibson assembly are listed in Table S1 in the supplemental material.

### Genetic manipulation of C. parvum to generate stable transgenic lines.

Cryptosporidium parvum oocysts used in this study included the AUCP-1 isolate (kind gift from Mark Kuhlenschmidt’s laboratory, University of Illinois-Urbana Champaign), and IOWA-II strain purchased from Bunch Grass Farm (Deary, ID). The AUCP-1 isolate was used to generate the CDPK1-HA, CDPK1-KO, and TK-KO transgenic parasites, while the Bunchgrass IOWA-II strain was used to generate the CDPK1-HA-DDD line. The use of these isolates was based on availability of fresh oocysts, and both can be successfully used to make transgenic strains and with no observed differences in mouse infection experiments. Oocysts were excysted, and the sporozoites were electroporated with the respective Cas9/guide plasmid (50 μg) and repair DNA template (50 μg) using Lonza Nucleofector 4D device as described previously (29, 60). The transfected sporozoites were suspended in sterile phosphate-buffered saline (PBS) and delivered to IFN-γ KO mice via oral gavage. The stomachs of the mice were buffered twice with 8% sodium bicarbonate before gavage with sporozoites. Mice were administered paromomycin (16 mg/ml) in drinking water to select for neomycin-resistant parasites. For generating CDPK1-HA-DDD stable transgenic parasites, a combination of paromomycin (16 mg/ml) and trimethoprim lactate (Gemini Bio, Sacramento, CA) in drinking water was administered after 24 h of sporozoite delivery. Different concentrations (0.5 mg/ml or 2 mg/ml) of TMP in drinking water were tested for creating a stable transgenic parasite. The bottle was changed every 3 days to ensure stability of TMP in drinking water. Oocysts were purified from fecal material using sucrose flotation, followed by cesium chloride purification as described previously (60, 61). To passage the transgenic lines, feces from infected mice or purified oocysts were used to infect new IFN-γ KO mice (5 to 6 weeks old). Fecal material was collected for purification of enough oocysts for further experiments.

### Fecal luminescence assay.

Luciferase activity was measured directly from pooled fecal material collected from mouse cages to monitor the growth of parasites using the method developed previously with minor modifications (29). For fecal collection, infected mice were moved to the collection cage for 6 to 8 h; the collection cage contains a wired floor insert and a wet paper towel beneath the wired floor. Collected fecal material was stored at 4°C until the luminescence assay was performed. Pooled feces were mashed with the help of a sterile pipette tip, and 20 mg of material was aliquoted into a new microcentrifuge tube. To this tube, 10 to 15 glass beads were added, followed by addition of 1 ml lysis buffer (50 mM Tris-HCl [pH 7.6], 2 mM dithiothreitol [DTT], 2 mm EDTA, 10% [vol/vol] glycerol, and 1% Triton X-100). Lysis was done by vortexing at high speed for 1 min, followed by centrifugation at 8,000 rpm for 15 s. After centrifugation, 100 μl of the supernatant was added to three wells on a 96-well white plate, and 100 μl of Nanoluc substrate diluted in 1:50 Nanoluc lysis buffer (Promega, Madison, WI) was added to each well. Luminescence was measured on the Wallac Victor2 1420 multilabel counter (Perkin Elmer Inc.).

### PCR validation of transgenic lines.

Fecal genomic DNA was extracted from 100 mg of fecal material using a ZR Fecal DNA Miniprep kit (Zymo Research, Irvine, CA) following the manufacturer’s instructions. PCR was performed on extracted DNA to confirm the correct 5′ and 3′ integration events after homologous recombination. Sequences of the primers used to confirm integration for the CDPK1-KO, TK-KO, CDPK1-HA, and CDPK1-HA-DDD are listed in Table S1.

### Reverse transcription-quantitative PCR (RT-qPCR).

Host intestinal epithelial adenocarcinoma (HCT-8) cells were grown in RPMI medium with l-glutamine, 10% fetal bovine serum (FBS), 0.1 U/ml penicillin, 0.1 μg/ml streptomycin, 0.25 μg/ml amphotericin B, and 1 mM sodium pyruvate on a 24-well plate. The host cell medium was replaced with *Cryptosporidium* infection medium (RPMI containing 2% FBS, 0.1 U/ml penicillin, 0.1 μg/ml streptomycin, and 0.25 μg/ml amphotericin B) prior to infection. Wild-type oocysts (IOWA-II) were subjected to bleach treatment, washed in ice-cold PBS, and used for infecting the HCT-8 cells for 2, 6, 12, 24, 36, 48, and 72 h (three wells for each time point). Cell monolayer was scraped at each time point, and RNA was isolated from infected wells using the PureLink RNA minikit (Invitrogen). DNase I treatment was performed to remove any contaminating genomic DNA. cDNA was prepared using SuperScript III first-strand synthesis system (Invitrogen) following the manufacturer’s instructions. PCR was performed on the cDNA using PowerUp SYBR green master mix (Applied Biosystems) and primers for the *cdpk1* gene, C. parvum 18S rRNA (normalization control), and human actin on the ABI 7500 real-time PCR system. Relative expression of the *cdpk1* gene was calculated using the ΔΔ*C_T_* method, and expression was normalized to the mean expression of *cdpk1* at 2 h post-infection. Sequences of the primers used are listed in Table S1.

### Immunofluorescence assay.

HCT-8 cells were seeded onto six-well plates containing high-precision cover glasses, and grown to 60 to 70% confluence. Bleached and washed transgenic oocysts were used for infecting the HCT-8 monolayer for 24, 48, or 72 h. Cells were fixed with 4% paraformaldehyde in PBS, followed by permeabilization with 0.25% Triton X-100 in PBS. Blocking was performed with 1% bovine serum albumin (BSA) overnight. After blocking, cells were incubated with primary antibody for 1 h, followed by three washes with PBS. Primary antibodies used were anti-rat-HA (clone 3F10; Roche), mouse anti-5E3 (kind gift from David Sibley, Washington University School of Medicine), and rabbit anti-histone 3 acetyl Lys9 (anti-H3K9ac; Millipore Sigma). Fluorophore-conjugated secondary antibodies (Alexa Fluor 488, Alexa Fluor 568; Invitrogen) at a dilution of 1:500 was added, and cells were incubated for 1 h. Washing was performed three times with PBS, and Hoechst 33342 DNA stain was added during the first wash. Coverslips were inverted and mounted on glass slides using Vectashield antifade mounting medium (Vector Laboratories). Images were captured using super-resolution structured illumination microscopy (SR-SIM) on a Zeiss ELYRA S1 microscope. Z-stack images were collected and processed using automatic SR-SIM parameters in ZEN 2011.

### Western blotting.

Transgenic oocysts (3 × 10^6^ to 4 × 10^6^) were subjected to bleach treatment, washed with ice-cold PBS, and then used to infect HCT-8 cells grown in 24-well plates with or without TMP. After 48 h of infection, cells were harvested by scraping the monolayer. Samples were centrifuged at 13,000 rpm for 3 min, supernatant was discarded, and pellet was washed twice with PBS. The pellet was boiled in Laemmli sample buffer for 5 min and run on a 4 to 20% Tris-glycine-sodium dodecyl sulfate (SDS) Mini-Protean precast gel (Bio-Rad). After SDS-polyacrylamide gel electrophoresis (PAGE), the proteins were transferred onto a polyvinylidene difluoride (PVDF) membrane using Mini-Protean Trans-Blot Cell apparatus (Bio-Rad), followed by blocking overnight at 4°C in 4% non-fat milk. The membrane was incubated with a 1:1,000 dilution of primary antibody (anti-rat HA, clone 3F10) for 1 h, followed by three washes in PBS with 0.1% Tween 20 (PBST). This was followed by incubation with anti-rat horseradish peroxidase (HRP)-conjugated secondary antibody (1:20,000 dilution) for 1 h and multiple washes with PBST. The HRP was detected by chemiluminescence using the SuperSignal West Pico Plus chemiluminescent substrate (Thermo Fisher Scientific) on a FluorChem imager. the blot was stripped and reprobed with a 1:2000 dilution of mouse anti-CP23 (Lifespan Biosciences) and anti-mouse HRP-conjugated secondary antibody (1:20,000). Densitometric analysis of protein bands was performed in ImageJ.

### Histological examination.

For histological analysis, two cages of 5-week-old female IFN-γ KO mice (*n* = 5 per cage) were used for infection with CDPK1-HA-DDD oocysts (1,000 oocysts/mouse) via oral gavage. The mice in one cage were subjected to paromomycin and TMP treatment, while the mice in the other cage (control) were administered only paromomycin in drinking water. Fecal Nluc was measured every day to quantify infection. At day 11 post-infection, mice (*n* = 3) from both groups were euthanized, and the small intestine was resected. The distal end of the intestine was flushed with PBS and 10% neutral buffered formalin (Sigma, St. Louis, MO). Tissue samples were placed in histology cassettes, immersed for fixation in 10% formalin for 24 h, and then transferred to 70% ethanol until tissue processing and sectioning. The tissue samples were embedded in paraffin and transversely sectioned (4-μm sections), and slides were stained with hematoxylin-eosin (H&E). The slides were examined with an Olympus BX51 light microscope fitted with a DP70 camera, and images were captured at ×200 magnification for histopathological evaluation. ImageJ 1.52q was used to measure villus length and crypt depth for 30 villi (*n* = 10 villi per mouse) using the protocol described previously (62). The ratio of villus length to crypt depth was calculated, and a reduction in this ratio (<3:1 or 2:1) indicated partial to complete villus atrophy due to infection.

### *In vitro* drug assays.

HCT-8 cells were seeded onto 96-well plates and infected with C. parvum transgenic oocysts expressing Nluc for *in vitro* growth assay as described previously (19, 29). Briefly, purified transgenic Nluc-expressing oocysts (5,000 oocysts per well) were incubated with different concentrations of TMP or BKI-1294 (kind gift from Wesley Van Voorhis, University of Washington) for 48 h. No drug was added to the control wells. The culture supernatant was discarded from the wells after 48 h of incubation, and 100 μl of NanoGlo lysis buffer (Promega) was added to the wells and incubated for 15 min at 37°C and 5% CO_2_. The lysate was pipetted up and down several times, and 100 μl of NanoGlo lysis buffer containing 1:50 of NanoGlo substrate (Promega) was added to the wells. Lysates were transferred to white 96-well plates, and luminescence was measured on the Wallac Victor2 1420 multilabel counter (Perkin Elmer Inc.). Fifty percent effective concentration (EC_50_) values were calculated using a non-linear regression (curve fit of log dose versus response) in GraphPad Prism v8.

### Statistical analysis.

All statistical analyses were performed using GraphPad Prism v8. The comparison of growth (fecal luminescence) of CDPK1-HA transgenic parasite upon treatment with or without TMP was performed using non-parametric Mann-Whitney test. Multiple unpaired *t* tests were performed to compare differences in fecal luminescence measurements each day upon infection of mice with CDPK1-HA-DDD transgenic parasites and in the presence or absence of TMP in drinking water. Significant increase in parasite growth *in vitro* in the presence of different TMP concentrations was determined using two-way analysis of variance (ANOVA) with Tukey’s multiple-comparison test. Measurement of changes in small intestinal histology (villus length to crypt depth ratio) of infected mice and control mice was performed using one-way ANOVA with Dunnett’s multiple-comparison test. *P* < 0.05 was considered significant.
