# Supplementary material for: A Conditional Protein Degradation System To Study Essential Gene Function in Cryptosporidium parvum
Source: mBio. 2020 Aug 25;11(4):e01231-20. doi: 10.1128/mBio.01231-20 (PMC7448269; doi:10.1128/mBio.01231-20)
Supplement: TABLE S1 [file mBio.01231-20-st001.docx]

**Table S1: Sequences of oligonucleotides used in this study**

| **Region** | **Primer name** | **Sequence (5’ to 3’)** |
| --- | --- | --- |
| cdpk1_tagging guide | cdpk1_tag_g1F | GTTGgttcactactatttgagagt |
|  | cdpk1_tag_g1R | AAACactctcaaatagtagtgaac |
| cdpk1_KO guide | cdpk1KO_g1F | GTTGgtagcgggacctttgcagaa |
|  | cdpk1KO_g1R | AAACttctgcaaaggtcccgctac |
| Repair for cdpk1-HA tag and cdpk1-HA-DD tag | OH_CDPK1_HA3_tagF | tagactttaatgagtttgtagaaatgcttcagaattttgtcaggaatgaaCCTAGGTACCCGTACGACGTCCCGGAC |
|  | OH_CDPK1_HA3_tagR | taacaagactattgcctccctattgaaaataacaataatttagaactcggAATTAAGATAAAAAGAAAAACTTAATCGATAC |
| Repair for cdpk1-KO tag | OH_cdpk1_F | attttagttcaggaataatccaaacaactcacaattaaggcttgttataatggggaaactaaatatactgaaattcggtag |
|  | OH_cdpk1_R | gcctactctcaaatagtagtgaactctatatatttcaattctattattattaAATTAAGATAAAAAGAAAAACTTAATCGATACTATC |
| 5’ integration_cdpk1_HA | cdpk1_5'intF (P1) | caggcagatagcagtattcagatggaggagttggaatc |
|  | HA_rev (P2) | AGGATACGCATAATCGGGCACATCATAGGGATAGC |
| 3’ integration cdpk1_HA | 5290_neointF (P3) | CAGACTTAAGGCCCGTATGCCCGACGGTGAAGATCTTGTC |
|  | cdpk1_3'intR (P4) | cttggattttactcgaaccaatattataggggtacgactg |
| TK_gene | 5390_TKgeneF | ATGGCAAAATTATACTTTTACTATTCAGCAATGAATGC |
|  | 5391_TKgeneR | TTAGAAATTGTATTCTTCACAATTAATTATATGATGTTTTCTGC |
| Repair for TK-KO | OH_TKKO_F | ataatatcactcatacctactgcaaataaGATTGGAAATACTTTAATAAAtggggaaactaaatatactgaaattcggtag |
|  | OH_TKKO_R | tttttaggcactttcaagaggcgccatagctgcgccaaattttgcccgccTCAGAAGAATTCGTCAAGAAGACGATAGAAG |
| Nluc for integration check | Nluc_3810_R (P5) | CTTTGGATCGGAGTTACGGACAC |
| Gibson cloning of DDD | plicha_vec_F_gibson | TAACCCGGGATGCATCTTCATTTAG |
|  | plicHa_vec_R gibson | GGCATAATCTGGAACATCGTAAGG |
|  | DD_fwd gibson | acgatgttccagattatgccATCAGTCTGATTGCGGCGTTAGCG |
|  | DD_rev gibson | tgaagatgcatcccgggttaTCGCCGCTCCAGAATCTCAAAGCA |
| qPCR_cdpk1 | cdpk1_RTF | CACCAGTAGATGCCGTAGTAAAA |
|  | cdpk1_RTR | CCTCTCTCTCCTCCCATAGTT |
| qPCR_Cp18SrRNA | Cp18S_F | ATGACGGGTAACGGGGAAT |
|  | Cp18S_R | CCAATTACAAAACCAAAAAGTCC |
| qPCR_Human_Actin | HsActinF | TCCCTGGAGAAGAGCTACGAG |
|  | HsActinF | AGCACTGTGTTGGCGTACAGG |
