# Supplementary figures and images for: A Conditional Protein Degradation System To Study Essential Gene Function in Cryptosporidium parvum
Source: mBio. 2020 Aug 25;11(4):e01231-20. doi: 10.1128/mBio.01231-20 (PMC7448269; doi:10.1128/mBio.01231-20)

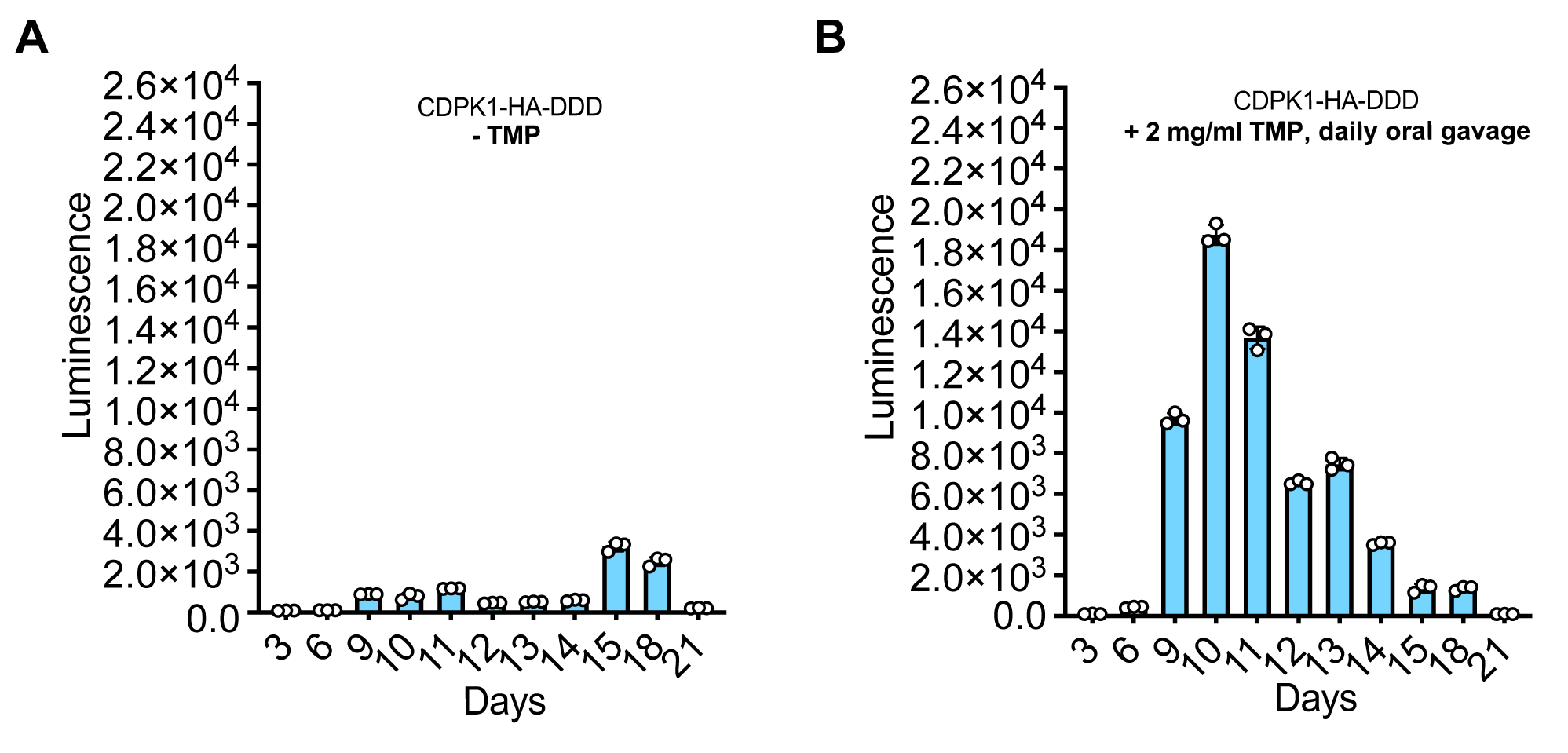

Supplement: FIG S1 [file mBio.01231-20-sf001.tif]

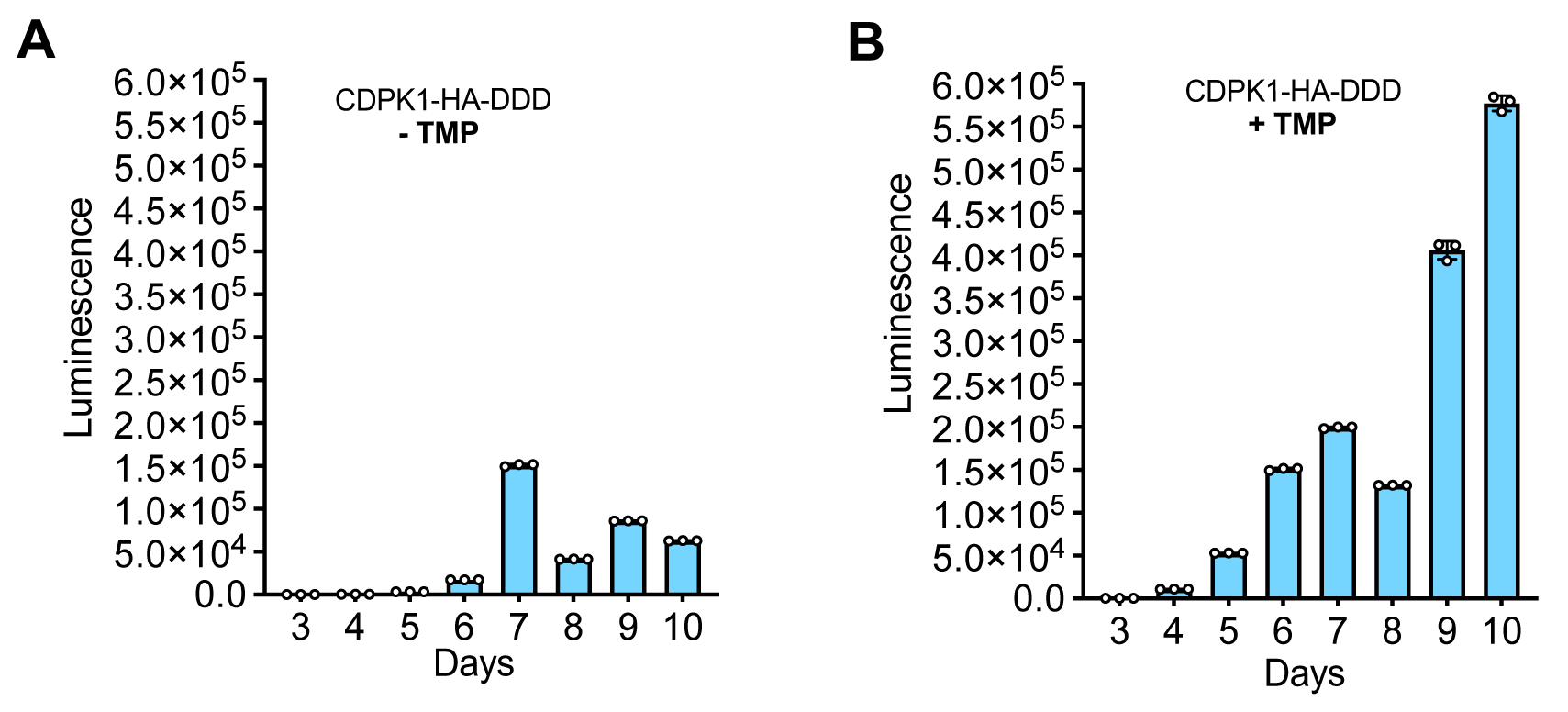

Supplement: FIG S2 [file mBio.01231-20-sf002.tif]
